# Variation in Physician Practice Styles within and across Emergency Departments

**DOI:** 10.1371/journal.pone.0159882

**Published:** 2016-08-12

**Authors:** Jessica Van Parys

**Affiliations:** 1 Economics Department, Hunter College, CUNY, New York, New York, United States of America; 2 The Dartmouth Institute for Health Policy and Clinical Practice, Geisel School of Medicine, Lebanon, New Hampshire, United States of America; Fraunhofer Research Institution of Marine Biotechnology, GERMANY

## Abstract

Despite the significant responsibility that physicians have in healthcare delivery, we know surprisingly little about why physician practice styles vary within or across institutions. Estimating variation in physician practice styles is complicated by the fact that patients are rarely randomly assigned to physicians. This paper uses the quasi-random assignment of patients to physicians in emergency departments (EDs) to show how physicians vary in their treatment of patients with minor injuries. The results reveal a considerable degree of variation in practice styles within EDs; physicians at the 75th percentile of the spending distribution spend 20% more than physicians at the 25th percentile. Observable physician characteristics do not explain much of the variation across physicians, but there is a significant degree of sorting between physicians and EDs over time, with high-cost physicians sorting into high-cost EDs as they gain experience. The results may shed light on why some EDs remain persistently higher-cost than others.

## Introduction

In recent years, policymakers have been looking for ways to improve the efficiency of the United States healthcare system. The idea is that a more efficient system would reduce healthcare costs for a given set of health outcomes. The focus on efficiency stems from the fact that per capita medical expenditures have increased dramatically in the US over the last 40 years relative to other OECD countries [1], while many national health indicators have not improved [2]. The popular press claims that 30% of US medical care is wasteful, so recent attention has turned to evaluating the costs and benefits of medical treatments [3] [4] [5] [6] [7] [8]. Since physicians are at the forefront of medical decision-making, policymakers are increasingly focused on physician decisions involving patient care, and recent research has attempted to understand why physicians might treat similar patients differently [9] [10] [11].

The challenge associated with characterizing physician practice styles is that, in most settings, patients select their physicians and physicians select their patients. If certain physicians always treat the healthiest patients, then their practice styles will look efficient, when in fact their practice styles reflect their patients’ underlying healthcare needs. This type of patient-physician sorting, however, is less likely to occur in emergency departments (EDs). Patients do not choose their physicians and physicians are required by law to treat all patients [12]. Therefore, EDs may provide more internally valid, quasi-experimental contexts in which to measure differences in physician practice styles.

This paper focuses on variation in ED physician practice styles and asks whether the variation can be explained by differences in physician characteristics, such as medical training or experience. Using data on all ED visits for patients with minor injuries in Florida from 2005 to 2011, where the standard of care ought to be relatively straightforward, I define ED physician practice styles using the total costs and the total number of procedures associated with ED physician caseloads. The ED data contain attending physician license numbers, which I match to Florida’s Healthcare Practitioner Database to obtain physician characteristics. The Practitioner Database contains information about medical school training, residency training, specialty, and gender. To estimate the variation in ED physician practice styles, I use physician fixed effect models that compare physicians who work in the same emergency departments, but at different points in time.

The paper has three findings. First, ED physician fixed effects explain an additional 3-11% of the variation in total costs and procedures for patients with minor injuries. Even when medical care should be similar for all patients, physicians still treat patients differently. Second, physician experience is the primary characteristic that is associated with practice style variation across physicians within EDs. Physicians with less than two years of experience spend 4.5% more per visit than physicians with more than six years of experience. Physicians with less than two years of experience do not generate fewer revisits to EDs, so their overall costs for “episodes of care” are higher than the overall costs of more experienced physicians. Third, physician experience is only associated with practice style variation because physicians differentially sort into and out of EDs over time. High-cost physicians are less likely to work in EDs over time, but if they remain working in EDs over time, then they are more likely to work in high-cost EDs. The results imply that physicians sort into institutions that reinforce their existing practice style patterns.

## Practice Style Variation Within and Across US Hospitals

There is a considerable amount of research that shows how healthcare delivery varies across hospitals and regions. After controlling for population health and local price levels, it finds significant geographical differences in medical reimbursements, hospitalizations, surgical procedures, and hospital readmissions for Medicare beneficiaries [13] [14] [15] [16]. These “regional variations” lead some to conclude that healthcare in the United States is “flat-of-the-curve” medicine [17]; i.e., that there are large differences in costs across regions, but there are fewer differences in observable health outcomes [18] [19] [20]. Potential explanations for high costs and flat outcomes include (1) “overuse,” where some hospitals prescribe invasive procedures on increasingly marginal patients for increased profit and (2) different production functions, where some hospitals are less productive than other hospitals at producing comparable outcomes [21].

Since much of the variation in healthcare delivery comes from variation in testing and treatment rather than price per se, a growing body of research focuses on the importance of physician practice styles and understanding why physicians treat similar patients differently. For example, Epstein and Nicholson (2009) find that within-region variation in cesarean section rates is two times greater than across-region variation, and that physician residency programs explain 4% of the within-region variation in cesarean section rates [9]. Doyle et al. (2010) randomize admitted patients to two resident teams in a Veterans Affairs hospital, where one residency team is affiliated with a higher-ranked medical institution. They find that the higher-ranked residency team produced 10-25% shorter and less expensive hospital stays with no differences in health outcomes [10]. Chan (2016) studies interns and residents in a Boston hospital and asks if the variation in physician practice styles is attributed to learning, authority, or differences in physician characteristics. He finds that interns learn and change practice styles as they advance to residency, but he finds no effects of other physician characteristics on practice style patterns [22]. These papers show that physician practice styles can vary within regions, and that medical training and experience can explain some of the differences in practice styles.

This paper makes two contributions to the literature on physician practice style variation. First, it is one of the first papers to focus on the practice styles of fully licensed medical doctors using a quasi-experimental design. Mehrotra et al. (2012) compare physicians across medical practices in Massachusetts and find that physicians with less than 10 years of experience have 13% higher cost profiles than physicians with more than 40 years of experience, and Chang et al. (2003) find that physicians with less than 6 years of experience spend more on laparoscopic-assisted vaginal hysterectomy, but no research has investigated the role of physician experience in settings where patient-physician selection is less likely to occur [23, 24]. Second, this is the first paper to show how physician sorting across institutions over time can explain associations between physician experience and practice styles. While Doyle (2010) and Chan (2016) have shown that physicians in training learn to adopt new practice styles, this paper extends their work by showing that physicians later sort into healthcare institutions that match their existing practice styles [10, 22].

## ED Visit Data and Physician License Data

Matching physicians to their patients is a necessary first step to characterize physician practice styles. Each time a patient visits a Florida ED she is treated by an attending physician on staff. The Florida ED data contain attending physician license numbers, which can be matched to the Florida Healthcare Practitioner Database. The Database contains the universe of medical and osteopathic physicians who have ever been licensed to practice medicine in Florida. It has information on each physician’s medical school, residency program, and personal characteristics. These data and this study have been approved by Columbia University’s Institutional Review Board protocol #AAAI1922 and are now approved by Hunter College’s Institutional Review Board protocol #2016-0018. Written informed consent was not given by participants. A waiver of consent was granted because the ED data were anonymized and de-identified prior to analysis.

This paper uses a sample of ED visits for patients with minor injuries to estimate the variation in ED physician practice styles. To identify patients with minor injuries, I use the primary diagnosis on the visit record and match it to the Healthcare Cost and Utilization Project’s (HCUP) Clinical Classification Software (CCS). The CCS diagnosis categories are more general than ICD-9-CM codes, so there is less scope for physicians to differentially “upcode” patients. Information about the CCS can be found in HCUP’s CCS user guide. To construct the sample, I keep patients who have injuries where the probability of hospitalization is less than 10%, though the average hospitalization rate for the sample is 1% (Table 1).

**Table 1:** Emergency Department Visits for Patients with Minor Injuries.

Patient’s Primary Diagnosis	Fraction of Sample	Hospitalization Rate	CCS Code
Joint disorders	0.020	0.021	225
Arm fracture	0.072	0.059	229
Sprains and strains	0.255	0.0008	232
Open wounds of head, neck, or trunk	0.094	0.011	235
Open wounds of extremities	0.137	0.010	236
Superficial injury or contusion	0.239	0.002	239
Burns	0.015	0.036	240
Poisoning from nonmedical substance	0.012	0.019	243
Other injuries from external causes	0.093	0.016	244
Lymphadenitis	0.006	0.028	247
Allergic reactions	0.058	0.003	253
N = # Visits	8,517,491	0.01	

Notes: Data source is the FL AHCA and HCUP Emergency Department Data. The diagnoses codes come from HCUP’s Clinical Classification System (CCS) chapter 16, titled “Injury and poisoning.” The HCUP CCS maps ICD-9-CM diagnosis codes to 260 aggregated diagnosis types. *N* is the number of ED visits for patients with minor injuries, where the minor injury is the primary diagnosis on the ED record. The hospitalization rate is calculated using the number of patients who were hospitalized with each injury divided by the total number of patients with each injury.

I focus on the sample of patients with minor injuries for two reasons. First, minor injuries are relatively straightforward to treat, so it would be unusual to detect large practice style variation across physicians treating these types of patients. Second, patients with minor injuries are rarely admitted to the hospital. This is an important feature because the Florida data do not contain identifying information about ED physicians when patients get admitted to the hospital. The ED physician can be inferred from the timing of the visit, but inference is imperfect because most hospitals have multiple ED physicians working at the same time. Therefore, to reduce the amount of measurement error that results from imperfect attribution of ED physicians to hospitalized patients, I focus on patients with minor injuries where 99% of the sample has identifying information about the ED physicians responsible for treatment and I attribute identifying information for ED physicians to the remaining 1% of the sample that gets hospitalized.

The summary statistics for the sample appear in Tables 1–3. Table 1 shows that there are eleven types of injuries that account for 8.5 million, or 21% of Florida ED visits from 2005 to 2011. Some of the most common injuries include arm fractures, sprains and strains, open wounds of the extremities, and superficial injuries or contusions. Tables 2 and 3 show how the sample of visits for patients with minor injuries compares to the sample of visits for patients without minor injuries. Patients with minor injuries are more likely to be male, to be white, to be younger, and to have private health insurance compared to patients who do not have minor injuries. The variation in the number of ED visits is relatively constant throughout the year, but there are more visits during the waking hours of 8am-12am.

**Table 2:** Characteristics of Patients with and without Minor Injuries.

	Minor Injuries		Otder Conditions	
Patient Characteristics	Mean	Standard Dev	Mean	Standard Dev
Female	0.48	0.50	0.57	0.50
Age	36.49	23.03	39.65	25.20
White	0.66	0.47	0.58	0.49
Black	0.18	0.38	0.23	0.42
Hispanic	0.13	0.33	0.16	0.37
Other Race	0.04	0.19	0.04	0.19
Private Insurance	0.34	0.47	0.25	0.43
Medicaid	0.18	0.38	0.25	0.43
Medicare	0.15	0.36	0.23	0.42
Uninsured	0.26	0.44	0.23	0.42
Other Insurance	0.08	0.27	0.03	0.18
Visit Timing	Mean	Standard Dev	Mean	Standard Dev
Quarter 1: Jan-March	0.24	0.43	0.26	0.44
Quarter 2: Apr-June	0.26	0.44	0.24	0.43
Quarter 3: July-Sep	0.26	0.44	0.25	0.43
Quarter 4: Oct-Dec	0.25	0.43	0.25	0.44
Weekend	0.30	0.46	0.28	0.45
Overnight: 12am-8am	0.12	0.33	0.17	0.37
Daytime: 8am-4pm	0.43	0.50	0.43	0.50
Evening: 4pm-12am	0.45	0.50	0.40	0.49
Healthcare Utilization	Mean	Standard Dev	Mean	Standard Dev
Total Costs	$630.17	$2,680.79	$2,529.79	$7,465.44
Radiology Costs	$244.48	$552.79	$443.19	$986.60
ED Costs	$194.89	$168.63	$250.45	$324.81
Laboratory Costs	$34.03	$289.53	$431.97	$1,084.88
Pharmacy Costs	$34.90	$798.77	$370.38	$2,273.12
Medical Devices Costs	$26.66	$397.91	$157.28	$1,016.80
Other Costs	$95.21	$1,255.24	$876.53	$3,420.52
Number of Procedures	3.37	2.42	3.79	3.60
Hospitalized	0.01	0.10	0.21	0.41
N = # Visits	8,517,491		40,077,076	

Notes: Data source is the FL AHCA Emergency Department Data.

**Table 3:** Characteristics of ED Physicians who Treat Patients with and without Minor Injuries.

	Minor Injuries		Other Conditions	
Physician Characteristics	Mean	Standard Dev	Mean	Standard Dev
Experience (years)	13.74	9.16	13.22	9.05
<2 Years of Experience	0.05	0.23	0.06	0.24
2–4 Years of Experience	0.08	0.27	0.09	0.28
4–6 Years of Experience	0.09	0.29	0.10	0.29
<6 Years of Experience	0.23	0.42	0.24	0.43
Degree of Medicine (M.D.)	0.80	0.40	0.82	0.38
Top-20 Medical School	0.05	0.22	0.05	0.22
US Medical School	0.77	0.42	0.74	0.44
Female Physician	0.19	0.39	0.19	0.39
Spanish-Speaking Physician	0.21	0.41	0.24	0.44
Specialist Physician	0.10	0.30	0.11	0.31
# Physicians	3,201		10,708	
# Visits	8,517,491		40,077,076	

Notes: Data sources include the FL AHCA Emergency Department Data and the FL Department of Health’s Healthcare Practitioner Database.

The average costs associated with ED visits for minor injuries appear in the bottom panel of Table 2. The average total cost per visit is $630, which is considerably lower than the total costs for patients without minor injuries ($2,530). Most of the total costs come from radiology fees ($244) and ED facility fees ($195). Costs are constructed by multiplying the hospital charge by the cost-to-charge ratio (CCR), where the CCR comes from HCUP. I use the hospital group CCR because it is available for all hospitals in the sample. The CCR varies across hospitals and years and it captures the share of charges that hospitals typically recoup from payers. Multiplying the charges by the CCR makes hospital costs more comparable across hospitals over time. On average, patients with minor injuries receive 3.4 procedures compared to 3.8 procedures for the sample of patients without minor injuries. Lastly, patients with minor injuries have a 1% probability of hospitalization compared to a 21% probability among patients without minor injuries.

Table 3 shows that there are 3,201 Florida ED physicians who treat on average 95 patients with minor injuries per quarter, compared with 10,708 ED physicians who treat on average 134 patients without minor injuries per quarter. There are 207 hospital emergency departments and most ED physicians work in multiple EDs. The average physician has about 14 years of experience post-residency. Twenty-three percent of physicians have less than 6 years of experience and 5% of physicians have less than two years of experience. Eighty percent of physicians hold Doctor of Medicine degrees (M.D.s) instead of Doctor of Osteopathic Medicine degrees (D.O.s), 19% are female, 21% speak Spanish, and 10% are specialists, where a specialist is certified in a field other than emergency medicine, internal medicine, family practice, or pediatrics. Most physicians attended medical school in the United States and five percent attended top-20 medical schools for primary care according to the 2013 US News and World Report rankings. I focus on the US News primary care rankings because those programs might be the best at training physicians to treat minor injuries in either a primary care or an ED setting. In general, the ED physicians who treat patients with minor injuries do not appear to be very different from the ED physicians who treat all other patients.

## Empirical Strategy

This paper’s conceptual experiment takes two patients with minor injuries who arrive in the same emergency department, but who receive different physician assignments. The question is whether their physician assignments affect the quality and quantity of healthcare services they receive. The empirical strategy proceeds in three parts. First I ask how much of the variation in healthcare utilization is explained by the attending ED physician. Conditional on the patient’s demographic characteristics, health insurance, hospital ED, and the approximate timing of the visit, do physician fixed effects improve the fit of the healthcare production function? Next I justify the quasi-experimental design. I show that conditional on the hospital and the approximate timing of the visit, patients are quasi-randomly assigned to ED physicians. Last I describe how I estimate the relationship between physician characteristics and physician practice styles.

### Variation in ED Physician Practice Styles

There is documented variation in healthcare utilization across regions, but variation in healthcare utilization within emergency departments has not been well studied. This section shows that such variation exists and that the variation is partially explained by physician fixed effects.

At the outset, it is not clear that patients who visit the same emergency department for the same injury would be treated differently. By design, two observably similar patients treated at the same ED at roughly the same time have the same healthcare needs and have the same access to facilities and ancillary staff. They have identical characteristics, so the hospital is unlikely to have an incentive to treat them differently. However, the patients can have different attending physician assignments and attending physicians may have different practice styles. So a useful first step is to determine whether attending physician fixed effects increase the goodness-of-fit in a model of healthcare utilization that already controls for the patient’s demographic characteristics, health insurance, hospital ED, and time period. To do this, I estimate the following two models on the ED physician panel data set described in the previous section,

$$\begin{array}{ccc} Log(Y_{ijht}) & = & \alpha +\gamma T_{t}+\kappa X_{i}+\delta_{h}+\lambda_{j}+\epsilon_{ijht} \end{array}$$ (1)

$$\begin{array}{ccc} Log(Y_{ijht}) & = & \alpha +\gamma T_{t}+\kappa X_{i}+\delta_{h}+\lambda_{jt}+\epsilon_{ijht} \end{array}$$ (2)

*Y*_*ijht*_ includes either the total ED costs or the total number of procedures for patient *i* of physician *j* in hospital *h* at time *t*. *T*_*t*_ is a vector of time fixed effects, including the year×quarter, the weekday, and the shift. There are three 8-hour shifts: shift 1 is from 12:00am to 8:00am, shift 2 is from 8:00am to 4:00pm, and shift 3 is from 4:00pm to 12:00am. *X*_*i*_ is a vector of patient characteristics and health insurance categories. Patient characteristics include gender, age, race, and ethnicity. Health insurance categories include Medicare, Medicaid, private insurance, other insurance, or self-pay. Sample means for the control variables are listed in Table 2. *δ*_*h*_ is a hospital fixed effect, *λ*_*j*_ is a physician fixed that does not vary across hospitals or time, and *λ*_*jt*_ is a physician-year-of-experience fixed effect that does not vary across hospitals, but does vary within physicians over discrete years of experience since residency (numbered 1, 2, …, up to more than 30 years). Eq (1) tests whether there are fixed differences across physicians in their practice styles (i.e., “between” differences). Comparing the goodness-of-fit from Eqs (1) and (2) tests whether there are also “within” differences in physician practice styles. If there is no meaningful variation in physician practice styles within EDs, then controlling for physician fixed effects or physician×experience fixed effects should not increase either model’s goodness of fit.

Results for model fit appear in Table 4. Column 1 begins by controlling for patient characteristics. Then each column adds an additional set of control variables. The focus is on the differences from columns 3-6 when hospital fixed effects, physician fixed effects, and physician×experience fixed effects are added to the model. Column 3 shows that hospital fixed effects explain 9% of the variation in total ED costs and procedures before controlling for physician fixed effects. Column 4 shows that physician fixed effects explain 10-11% of the variation in costs and procedures before controlling for hospital fixed effects. Incorporating physician fixed effects in addition to hospital fixed effects explains an additional 3% of the variation in total costs and procedures (comparing columns 4 and 5). The results suggest that physicians within hospitals treat similar patients differently, which is consistent with research in this area and points to a growing topic of exploration.

**Table 4:** Measuring Practice Style Variation using ED Physician Fixed Effects.

	(1)	(2)	(3)	(4)	(5)	(6)
Shift, Weekday, Year×Quarter FEs	X	X	X	X	X	X
+ Patient Characteristics		X	X	X	X	X
+ Hospital FEs			X		X	X
+ ED Physician FEs				X	X	
+ ED Physician×Experience FEs						X
Log(Total Costs): Adjusted *R* ^2^	0.03	0.08	0.17	0.19	0.20	0.22
Log(Total Procedures): Adjusted *R* ^2^	0.02	0.06	0.15	0.16	0.18	0.22

Notes: This table reports the adjusted *R*^2^s from regressions that capture the variation in healthcare utilization within and across EDs. The dependent variables are the Log(Total Costs) and the Log(Total #Procedures) for each ED visit. The sample includes ED visits for patients with minor injuries. The model in the first column controls for shift, weekday, and year×quarter fixed effects. The model in the second column adds patient age, gender, race, ethnicity, and insurance. The model in the third column adds hospital fixed effects. The model in the fourth column removes hospital fixed effects and adds physician fixed effects. The model in the fifth column includes physician and hospital fixed effects. The model in the sixth column includes physician×experience and hospital fixed effects.

**Fig 1:**
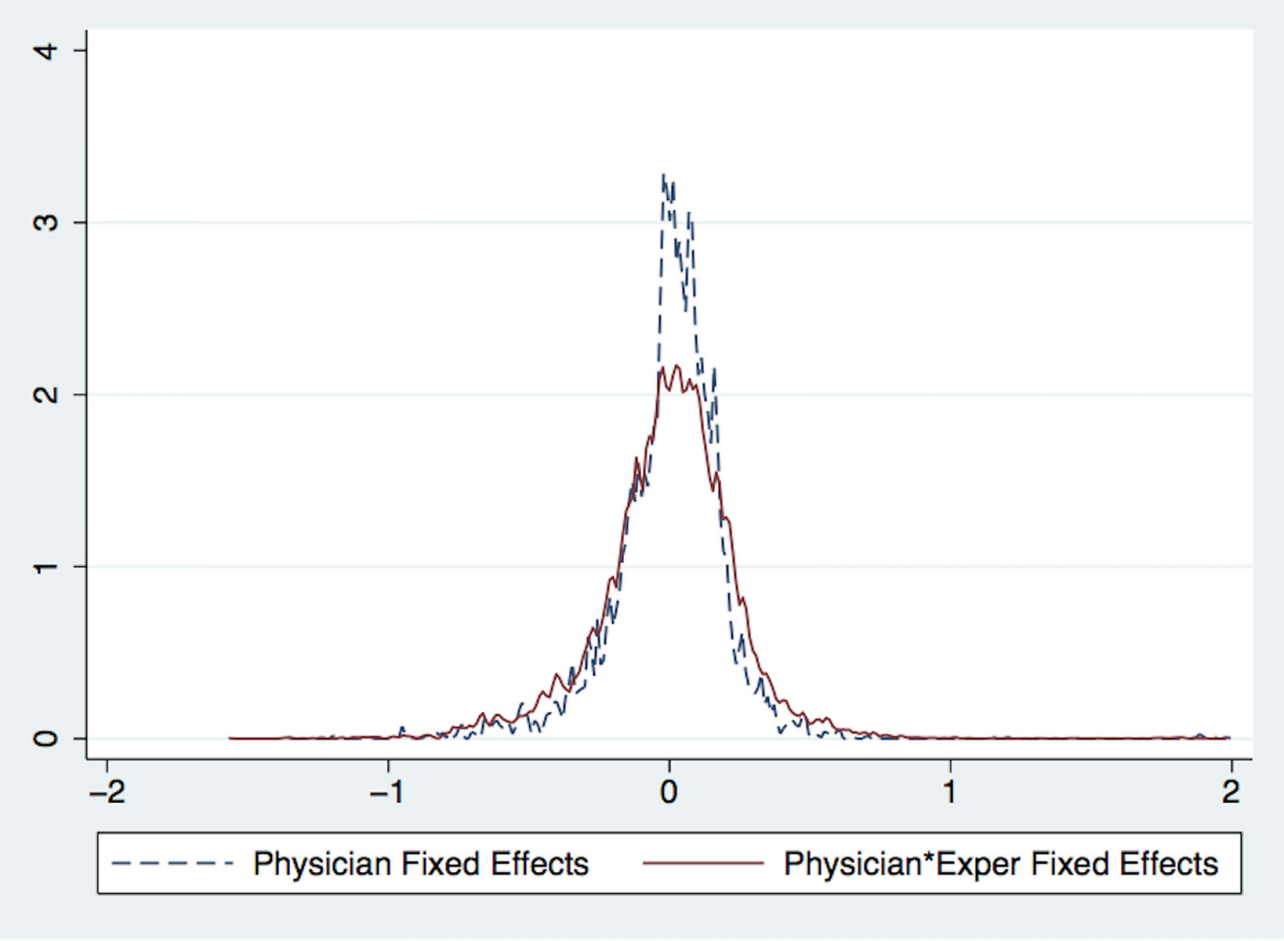
Distributions of Physician and Physician×Experience Fixed Effects for Log(Costs). Notes: This figure plots the distributions of physician fixed effects and physician×experience fixed effects. The blue dotted line plots physician fixed effects *λ*_*j*_ from Eq (1) and the red solid line plots physician×experience fixed effects *λ*_*jt*_ from Eq (2).

Not all of the variation in physician practice styles is fixed however; some of it is time-varying. For example, columns 5 and 6 show that physician×experience fixed effects explain an additional 2-4% of the variation in total costs and procedures compared to physician fixed effects alone. Fig 1 plots the distributions of physician fixed effects (*λ*_*j*_) and physician×experience fixed effects (*λ*_*jt*_) after adjusting the estimates using the empirical Bayesian shrinkage method described in Morris (1983) [25]. It shows that there is more variation in physician×experience fixed effects than physician fixed effects and a Kolmogorov-Smirnov test of distributional equivalence confirms with a p-value<0.0001. To understand the magnitude of the difference, the distribution of physician fixed effects shows that a physician in the 75th percentile spends 20% more than a physician in the 25th percentile, while the distribution of physician×experience fixed effects shows that a physician in the 75th percentile spends 26% more than a physician in the 25th percentile.

### Quasi-Random Assignment of Patients to ED Physicians

One threat to the experimental design is the systematic sorting of patients to physicians. With sorting, patient characteristics can be correlated with physician characteristics and practice styles. For instance, experienced physicians may perform more procedures if they treat patients who have more complicated medical conditions. Sorting between patients and physicians makes it difficult to uncover the association between physician characteristics and practice styles. To address this concern, I use the following equation to test whether there is sorting between patients and physicians in emergency departments,

$$\begin{array}{ccc} P_{jt} & = & \alpha +\gamma T_{t}+\kappa X_{i}+\delta_{h}+\epsilon_{ijht} \end{array}$$ (3)

*P*_*jt*_ is one of the physician characteristics listed in Table 3 for physician *j* who treats patient *i* in hospital *h* at time *t*. *P*_*jt*_ may represent physician experience, whether the physician has a M.D. degree, graduated from a top-20 medical school, attended medical school in the US or abroad, is female, is a specialist, or speaks Spanish. Physician experience is calculated as the difference between the patient’s visit-date and the physician’s residency completion date. The rest of the variables in Eq (3) are the same as in Eqs (1) and (2). The purpose of estimating Eq (3) is to test whether some types of patients are more likely to be treated by some types of physicians.

If patient-physician assignments are quasi-random within the ED, then patient characteristics should not predict physician characteristics in Eq (3). The *κ* vector tests whether patient characteristics are correlated with attending physician characteristics. If the coefficients within *κ* differ significantly from zero, then patients and physicians sort on observable characteristics, which can invalidate the quasi-experimental design. The standard errors for Eq (3) are clustered at the physician-level.

The results for Eq (3) appear in Table 5, and they show that patient characteristics explain very little of the physician assignment. Individual patient characteristics are uncorrelated with physician characteristics in 52 out of 63 cases. In the eleven cases where the characteristics are statistically significantly correlated, the coefficient estimates are very small and marginally significant. Moreover, 3 out of 63 cases should be significant with a p-value = 0.05. Therefore, patient-physician sorting within EDs is unlikely to drive much of the results from the previous section. Nevertheless, because some patient characteristics are correlated with some physician characteristics, I next explore the degree to which the results from the previous section might be biased due to sorting between patients and physicians within EDs.

**Table 5:** Quasi-Random Assignment of Patients to ED Physicians.

	(1)	(2)	(3)	(4)	(5)	(6)	(7)
	Exp	M.D.	Top-20	US	Female	Spanish	Specialist
Female	0.0139	-0.0002	0.00004	0.0005	0.0009*	0.0002	-0.0007*
	(0.0084)	(0.0004)	(0.0003)	(0.0004)	(0.0004)	(0.0004)	(0.0003)
Age	0.0011	-0.0001	0.00003	0.0003**	-0.0003**	-0.00001	0.0002***
	(0.0018)	(0.0001)	(0.0001)	(0.0001)	(0.0001)	(0.0001)	(0.00004)
Black	0.0110	-0.0017*	-0.0012	0.0002	0.0011	0.0009	-0.0009
	(0.0207)	(0.0008)	(0.0007)	(0.0010)	(0.0009)	(0.0009)	(0.0008)
Hispanic	0.0078	0.0015	-0.0021*	-0.0017	0.0027	0.0006	-0.0019
	(0.0519)	(0.0020)	(0.0009)	(0.0022)	(0.0019)	(0.0020)	(0.0011)
Other Race	0.0604	0.0013	0.0037	0.0005	0.0034	0.0018	0.0028
	(0.0785)	(0.0028)	(0.0022)	(0.0035)	(0.0031)	(0.0036)	(0.0026)
Medicaid	0.0452	0.0011	-0.0014	-0.0045**	0.0016	-0.0016	-0.0010
	(0.0327)	(0.0014)	(0.0008)	(0.0016)	(0.0014)	(0.0014)	(0.0009)
Medicare	-0.0417	0.0003	-0.0007	-0.0056*	0.0060*	-0.0025	-0.0037**
	(0.0493)	(0.0020)	(0.0013)	(0.0028)	(0.0029)	(0.0028)	(0.0012)
Self-Pay	0.0060	-0.0003	-0.0005	0.0011	-0.0017	0.0007	0.0013
	(0.0258)	(0.0010)	(0.0008)	(0.0013)	(0.0012)	(0.0012)	(0.0008)
Other Insurance	-0.0352	-0.0001	0.0007	-0.0001	-0.0006	0.0006	0.0013
	(0.0318)	(0.0013)	(0.0007)	(0.0016)	(0.0017)	(0.0016)	(0.00112)
Hospital FEs	Y	Y	Y	Y	Y	Y	Y
Year×Quarter FEs	Y	Y	Y	Y	Y	Y	Y
Shift, Weekday FEs	Y	Y	Y	Y	Y	Y	Y
N	8,517,491	8,517,491	8,517,491	8,517,491	8,517,491	8,517,491	8,517,491
*R*^2^	0.15	0.12	0.09	0.16	0.10	0.15	0.10

Notes: The dependent variables are physician characteristics and they are listed at the top of each column. The independent variables are patient characteristics and they are listed as rows. Each column represents estimates from a different regression. The sample includes ED visits for all patients with minor injuries in Florida from 2005 to 2011. All regressions control for hospital fixed effects, year×quarter fixed effects, weekday fixed effects, and shift fixed effects. Standard errors are clustered at the physician-level and are reported in parentheses.

*p < 0.05

**p < 0.01

***p < 0.001

**Fig 2:**
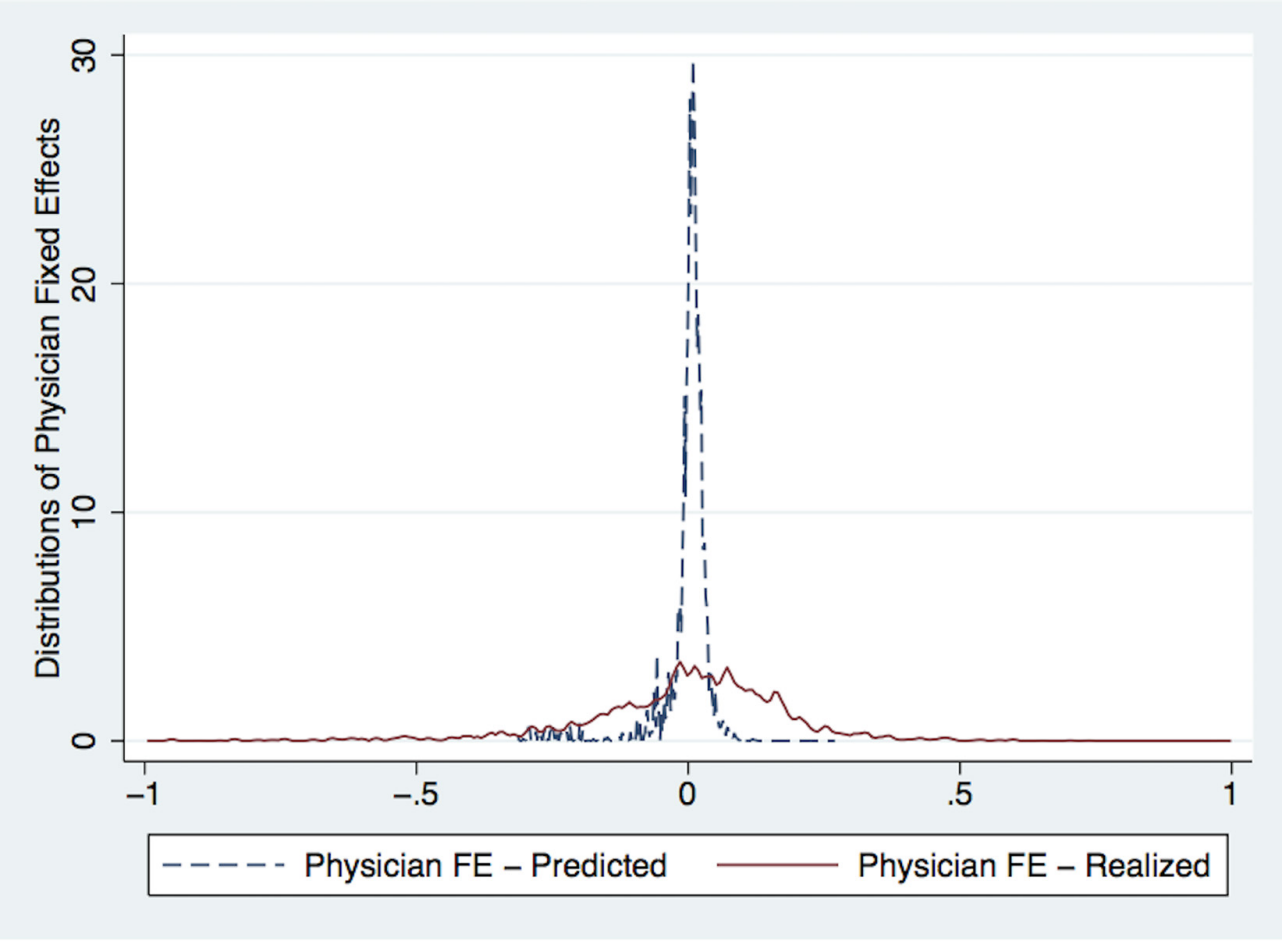
Distributions of Physician Fixed Effects for Predicted vs. Realized Log(Costs). Notes: This figure plots the distributions of physician fixed effects for predicted versus realized Log(Total Costs). The blue dotted line plots the Bayesian-shrinkage physician fixed effects for predicted Log(Total Costs), where Log(Total Costs) were predicted using patient age, sex, race, ethnicity, and insurance. The red solid line plots the Bayesian-shrinkage physician fixed effects from Eq (1).

To estimate how the results may be biased, I predict total costs using patient characteristics (age, sex, race, ethnicity, insurance) and regress the predicted values on hospital fixed effects, year×quarter fixed effects, weekday fixed effects, shift fixed effects, and physician fixed effects. The F-test on the physician fixed effects yields a value of 72.31, so I can reject the null hypothesis that there is no sorting between ED patients and physicians. To calibrate the degree of bias due to sorting on observable characteristics, I first plot the distribution of the Bayesian-shrinkage physician fixed effects that result from the latter regression. Then I compare that distribution to the distribution of Bayesian-shrinkage physician fixed effects from column 5 in Table 4. If sorting on observables explained most of the variation across physicians in their spending patterns, then those distributions would overlap considerably. If, however, there is a relatively small degree of sorting and there is meaningful variation across physicians in their spending patterns, then we would expect the first distribution to be clustered around zero with small variance and the second distribution to be centered at zero, but to exhibit much higher variance. Fig 2 plots these two distributions and supports the second interpretation of the data. Fig 2 also shows that physicians near the 75th percentile of the predicted costs distribution treat patients that are only 2% more costly than the patients treated by physicians at the 25th percentile of the distribution.

Second, I ask how likely it is that a “high-cost” physician, calculated using the Bayesian-shrinkage physician fixed effects reported in column 5 of Table 4, matches to a predictably “high-cost” patient, calculated using the observable patient characteristics age, sex, race, ethnicity, and insurance. The results appear in Table 6 and show that a one-standard deviation increase in predicted patient costs yields a 0.047-standard deviation increase in the Bayesian-shrinkage physician fixed effects (i.e., “Physician Costs”). The *R*^2^ in Table 6 also shows that predicted patient costs can only explain about 0.2% of the variation in physician costs. Therefore, sorting between patients and physicians is unlikely to be very problematic in the ED context.

**Table 6:** Correlation Between Predicted Patient Costs and Physician Fixed Effects.

	Physician Cost (I.e., Physician FE)
Predicted Patient Cost	0.0467** (0.0073)
N	8,517,491
*R*^2^	0.0022

Notes: Predicted Patient Costs are estimated from a regression of Log(Total Costs) on patient age, sex, race, ethnicity, and insurance. Physician Cost represents the Bayesian-shrinkage physician fixed effects from Eq (1). Predicted Patient Cost and Physician Cost are standardized to have mean = 0 and variance = 1. Standard errors are clustered at the physician-level and are reported in parentheses.

*p < 0.05

**p < 0.01

***p < 0.001

### Physician Characteristics and Practice Styles

This section presents an ordinary least squares (OLS) model to estimate the relationship between physician characteristics and ED physician practice styles. The OLS model starts with the same healthcare production function as in previous sections, but replaces the physician fixed effects with a full set of observable physician characteristics. In this framework, physician experience varies within physicians over time, while physician medical training and personal characteristics are fixed within physicians over time. Thus, the estimating equation becomes,

$$\begin{array}{ccc} Log(Y_{ijht}) & = & \alpha +\beta_{1}1\left[exp_{jt}<2\right]+\beta_{2}1\left[2\leq exp_{jt}<4\right]+\beta_{3}1\left[4\leq exp_{jt}<6\right]\\ & & +\psi P_{j}+\gamma T_{t}+\kappa X_{i}+\delta_{h}+\epsilon_{ijht} \end{array}$$ (4)

*Y*_*ijht*_ includes either the total ED costs or the total number of procedures for each ED visit, depending on the specification. *T*_*t*_, *X*_*i*_, and *δ*_*h*_ are defined as in previous sections. *P*_*j*_ is a vector of physician characteristics that do not vary within physicians over time. These characteristics include the physician’s gender, specialty, Spanish-speaking ability, medical degree type, US medical school training, and whether the physician’s medical school is ranked in the top-20. To estimate the effects of physician experience, I create indicator variables that equal 1 if the physician has less than two years of experience, two-to-four years of experience, or four-to-six years of experience post-residency. Therefore, *β*_1_, *β*_2_, and *β*_3_ should be interpreted relative to physicians with more than six years of experience. As in previous sections, the standard errors are clustered at the physician-level.

## Results

This paper asks whether ED physicians vary in their practice styles and whether physician characteristics can explain some of that variation. In this section, I find that physician experience and specialty explain differences in ED practice styles, but that medical education, gender, and Spanish-speaking ability do not. Physician experience is associated with practice styles primarily because high- and low-cost physicians sort into different work environments over time.

### ED Physician Characteristics and Healthcare Utilization

Table 7 shows how different physician characteristics are associated with total costs and procedures. Columns 1 and 4 of Table 7 report results from an OLS regression that does not control for patient characteristics, while columns 2 and 5 report results that control for patient characteristics. Comparing the estimates across the two sets of columns reveals the degree to which the estimates would have been biased due to sorting between physicians and patients. In general, the association between physician characteristics and practice styles is attenuated by controlling for patient characteristics, suggesting a small amount of bias due to nonrandom assignment; however, the results across the two sets of columns are not statistically different from one another and so the potential for bias is somewhat limited, consistent with what was found in the previous section.

**Table 7:** ED Physician Characteristics and Healthcare Utilization.

	(1)	(2)	(3)	(4)	(5)	(6)
	Log(Costs)	Log(Costs)	Log(Costs)	Log(Procs)	Log(Procs)	Log(Procs)
<2 Years of Experience	0.052***(0.012)	0.045***(0.010)	0.023(0.018)	0.039***(0.009)	0.034***(0.009)	0.012(0.019)
2–4 Years of Experience	0.046***(0.010)	0.039***(0.010)	0.012(0.014)	0.016*(0.008)	0.012(0.007)	-0.007(0.016)
4–6 Years of Experience	0.029**(0.010)	0.026**(0.009)	-0.003(0.010)	0.018*(0.008)	0.016*(0.007)	-0.003(0.012)
Degree of Medicine (MD)	-0.010(0.011)	-0.009(0.010)		-0.017*(0.007)	-0.017**(0.006)	
Top-20 Medical School	0.001(0.018)	-0.001(0.017)		0.010(0.011)	0.008(0.010)	
US Medical School	0.023*(0.012)	0.014(0.011)		-0.004(0.007)	-0.010(0.006)	
Female Physician	-0.0002(0.011)	0.008(0.010)		-0.006(0.007)	-0.001(0.006)	
Spanish-Speaking	-0.002(0.011)	-0.002(0.010)		0.005(0.007)	0.005(0.006)	
Specialist	0.038*(0.015)	0.029*(0.015)		0.019*(0.010)	0.014(0.009)	
Hospital FEs	Y	Y	Y	Y	Y	Y
Year×Quarter FEs	Y	Y	Y	Y	Y	Y
Shift, Weekday FEs	Y	Y	Y	Y	Y	Y
Patient Characteristics		Y	Y		Y	Y
Physician FEs			Y			Y
N	8,517,491	8,517,491	8,517,491	8,517,491	8,517,491	8,517,491
*R*^2^	0.12	0.17	0.20	0.11	0.15	0.18

Notes: The dependent variables are the log(Total Costs) and the log(Total Procedures) for each ED visit. The sample includes ED visits for all patients with minor injuries in Florida from 2005 to 2011. All regressions control for hospital fixed effects, year×quarter fixed effects, weekday fixed effects, and shift fixed effects. Columns (2), (3), (5), and (6) also control for patient age, gender, race, ethnicity, and health insurance. Columns (3) and (6) control for physician fixed effects. Standard errors are clustered at the physician-level and are reported in parentheses.

*p < 0.05

**p < 0.01

***p < 0.001

Table 7 shows that physician experience is the main characteristic that is associated with variability in total costs and procedures. Physicians with less than two years of experience post-residency spend 4.5% more per visit and they perform 3.4% more procedures than physicians with six or more years of experience. Physicians with 2-4 years of experience spend 3.9% more per visit than physicians with six or more years of experience, but there is no statistically significant difference in the number of procedures they perform. The experience effect tapers off as physicians age because physicians with 4-6 years of experience only spend 2.6% more per visit than physicians with more than 6 years of experience.

Several other physician characteristics are not associated with differences in ED practice styles. For example, physicians who trained at top-20 medical schools do not spend less than physicians who trained at lower-ranked medical schools. Similarly, US medical school training, physician gender, and Spanish-speaking ability do not explain differences in ED practice styles. Specialists spend 2.9% more than non-specialists, but they do not perform more procedures. Physicians with Doctor of Medicine degrees (M.D.s) perform 2% fewer procedures, but do not spend less than physicians with Doctor of Osteopathic Medicine degrees (D.O.s). Moreover, observable physician characteristics explain very little of the overall variation in total spending or procedures, which can be seen by comparing the *R*^2^ in columns 2 and 5 of Table 7 to the *R*^2^s in column 3 of Table 4. However, it is important to note that these lack of findings could be specific to the sample of patients with minor injuries.

Doyle et al. (2010) find that physicians who spend more on hospitalized patients tend to utilize more testing equipment than physicians who spend less, so Table 8 shows how physicians with less experience utilize different resources within the ED [10]. It shows that physicians with less experience spend more on all items, including but not limited to radiology tests, laboratory tests, and pharmacy prescriptions. Physicians with less than two years of experience spend $55.25 more per patient, where 65% of that higher spending comes from radiology testing, laboratory testing, and pharmaceutical prescriptions. Other types of spending account for the remaining 35%.

**Table 8:** ED Physician Experience and Itemized Spending.

	(1)	(2)	(3)	(4)	(5)	(6)	(7)
	Total	Radiology	Labs	Pharmacy	Devices	Facility	Other
<2 Years of Experience	$55.25***	$26.39***	$4.68***	$5.23**	$3.74**	$4.13*	$11.09**
	(10.23)	(4.81)	(0.97)	(1.77)	(1.14)	(1.70)	(3.93)
2–4 Years of Experience	$44.83***	$23.00***	$3.49***	$4.11**	$2.02*	$3.88*	$8.33**
	(8.29)	(4.40)	(0.82)	(1.32)	(0.85)	(1.61)	(3.32)
4–6 Years of Experience	$30.08***	$13.43***	$2.83***	$3.01*	$1.27	$0.37	$9.17**
	(7.60)	(3.83)	(0.75)	(1.22)	(0.70)	(1.69)	(2.91)
Dependent Variable Mean	$630	$244	$34	$35	$27	$195	$95
Hospital FEs	Y	Y	Y	Y	Y	Y	Y
Year×Quarter FEs	Y	Y	Y	Y	Y	Y	Y
Shift, Weekday FEs	Y	Y	Y	Y	Y	Y	Y
Patient Characteristics	Y	Y	Y	Y	Y	Y	Y
Physician Characteristics	Y	Y	Y	Y	Y	Y	Y
N	8,517,491	8,517,491	8,517,491	8,517,491	8,517,491	8,517,491	8,517,491
*R*^2^	0.02	0.08	0.02	0.004	0.01	0.20	0.06

Notes: The dependent variables are itemized costs for each ED visit. The sample includes ED visits for all patients with minor injuries in Florida from 2005 to 2011. All regressions control for hospital fixed effects, year×quarter fixed effects, weekday fixed effects, shift fixed effects, physician characteristics, and the patient characteristics: age, sex, race, ethnicity, and insurance. Standard errors are clustered at the physician-level and are reported in parentheses.

*p < 0.05

**p < 0.01

***p < 0.001

One unanswered question is whether physicians learn to spend less over time or whether high-cost physicians sort into new work environments over time. Columns 3 and 6 from Table 6 test the hypothesis that physicians learn to spend less over time by showing the relationship between physician experience and practice styles after controlling for physician fixed effects. The results show that physician experience is not associated with lower spending within physicians over time, suggesting that ED physicians do not learn to spend less over time.

**Fig 3:**
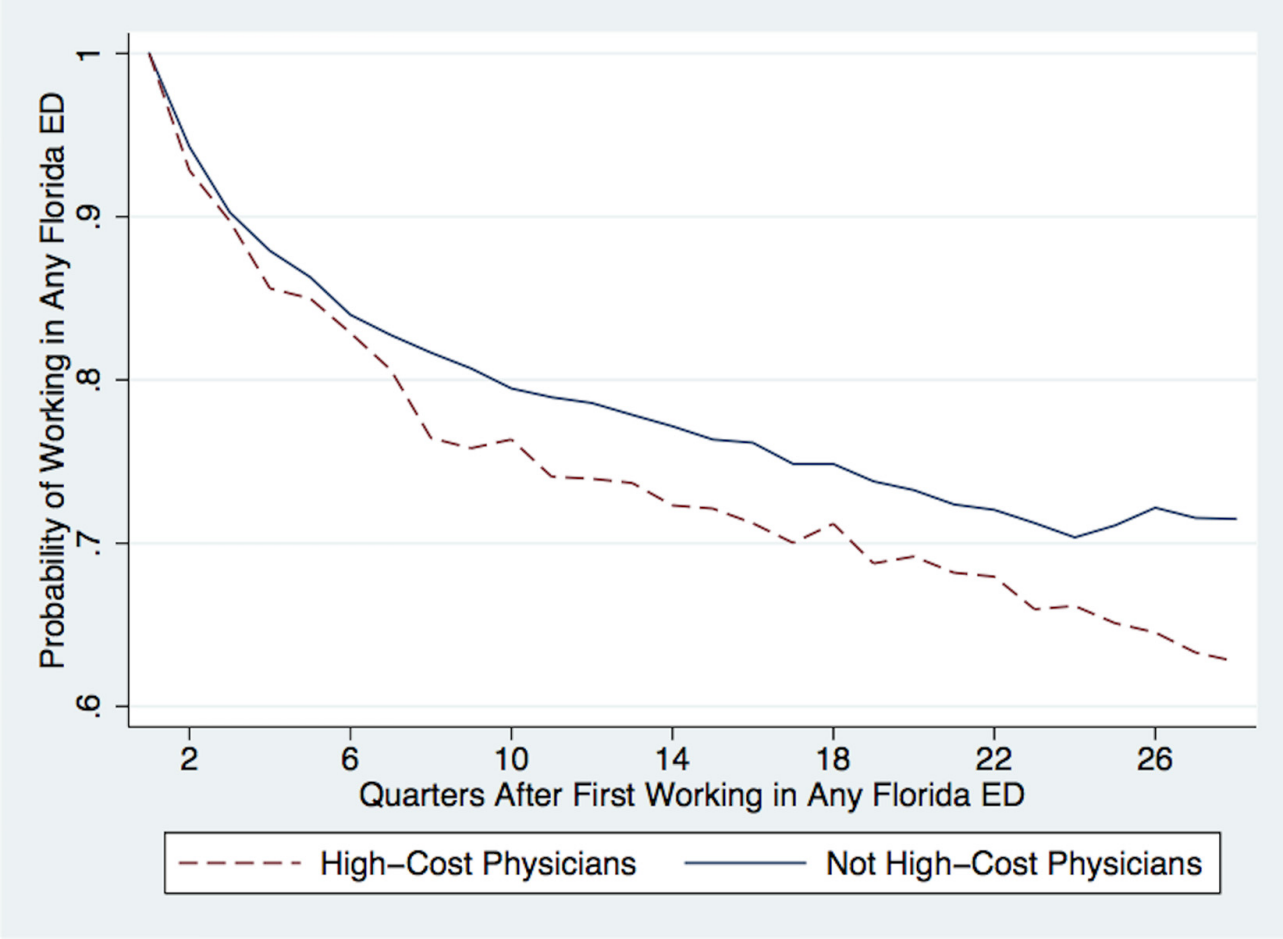
Attrition of High-Cost ED Physicians Over Time. Notes: This figure plots the probabilities that physicians work in any ED following their first quarter of work, where their earliest start date occurs on or after Q1-2005. The blue solid line plots the probability that physicians who are not high-cost work in any ED, while the red dotted line plots the probability that high-cost physicians work in any ED. High-cost physicians are in the top 10% of the Bayesian-shrinkage physician fixed effects distribution for Log(Costs) estimated from Eq (1).

**Fig 4:**
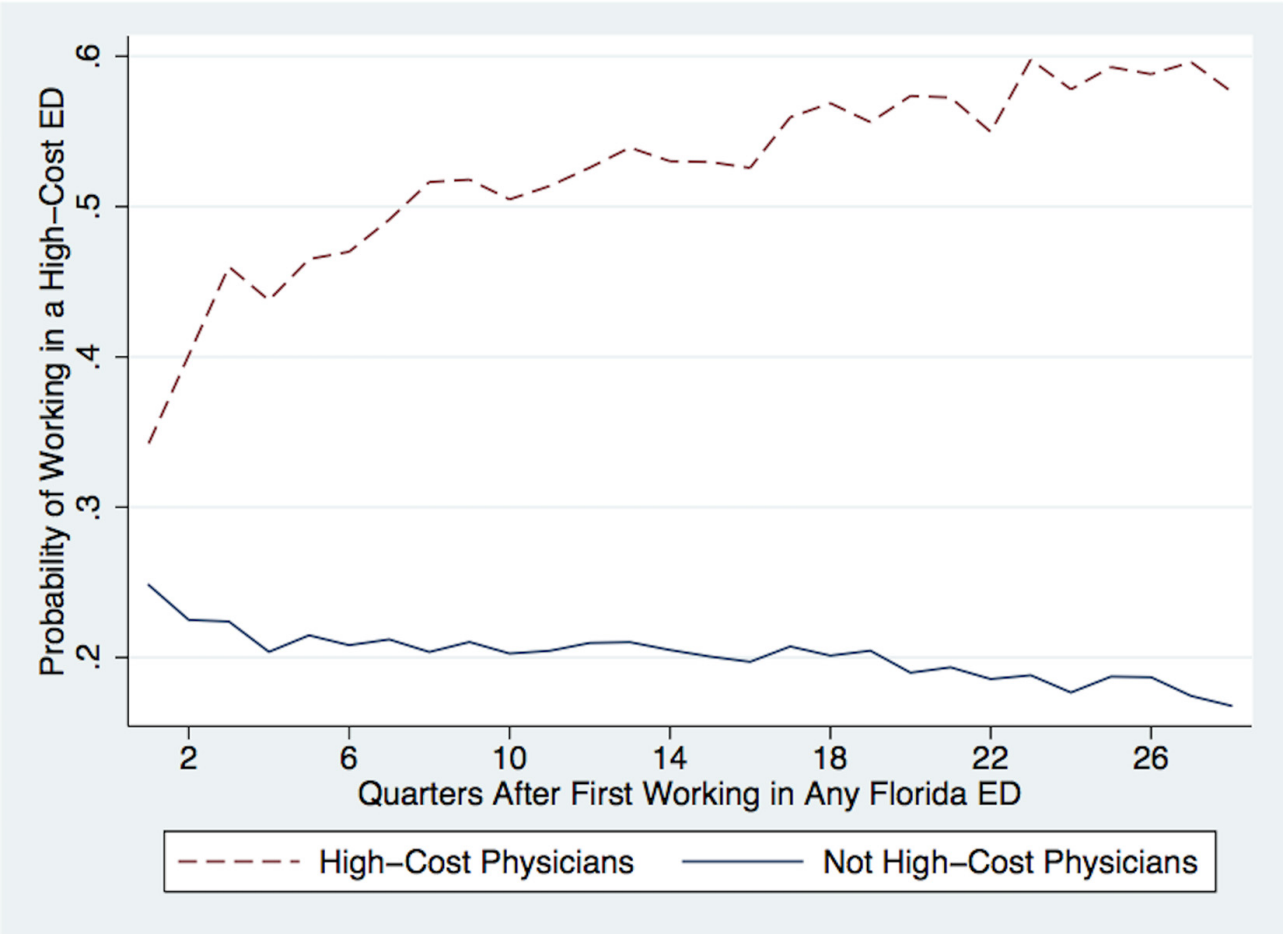
Sorting Between Physicians and EDs Over Time. Notes: This figure plots the probabilities that physicians work in high-cost EDs following their first quarter of work, where their earliest start date occurs on or after Q1-2005. The blue solid line plots the probability that physicians who are not high-cost work in a high-cost ED, while the red dotted line plots the probability that high-cost physicians work in high-cost EDs. High-cost physicians are in the top 10% of the Bayesian-shrinkage physician fixed effects distribution for Log(Costs) estimated from Eq (1). High-cost EDs are in the top 25% of the distribution for the average total costs of ED visits, where the average total costs are calculated using the entire sample of patients from Table 2.

Figs 3 and 4 test the hypothesis that high-cost physicians sort into new work environments over time. Physicians are considered “high-cost” if they are in the top 10% of the physician fixed effects distribution for Log(Costs) estimated from Eq (1). The x-axes in Figs 3 and 4 show quarters since physicians began working in any Florida ED, where the earliest possible start date was Q1-2005 because that is the start of the sample. The y-axis in Fig 3 shows the probability that physicians are still working in any Florida ED. The red dotted line represents high-cost physicians and the blue solid line represents all other physicians. The figure shows that high-cost physicians are 3% less likely to work in any Florida ED two years after their initial start dates.

Fig 4 shows that when high-cost physicians continue to work in Florida EDs, they are more likely to sort into high-cost EDs over time. A high-cost ED is an ED in the top quartile of the distribution for the average total costs of ED visits, where the average total costs are calculated using the entire sample of patients (from Table 2), not only the sample of patients with minor injuries. Within the first six years of experience post-residency, high-cost physicians are three times more likely to work in high-cost Florida EDs compared to non-high-cost physicians, and most of the sorting occurs within the first two years post-residency. Thus, experienced ED physicians only appear to have lower-cost practice styles. In reality, they have already sorted into work environments that reflect their styles.

### ED Physician Characteristics and Patient Health Outcomes

Does lower ED spending mean that physicians are practicing more efficiently, or are they providing lower quality care? To answer this question, it is important to note that patients with minor injuries are unlikely to experience many serious negative health outcomes. For example, the health economics literature often uses inpatient mortality and delayed hospital admission as health outcomes, but these are inappropriate outcomes in this context because only 1% of patients with minor injuries get admitted to the hospital on the initial visit, and almost no one dies in the hospital from a minor injury. Therefore, the faster physicians can diagnose injuries, and treat them, the more efficient they are. However, physicians can still make mistakes, which would result in lower quality care.

One way to measure lower quality healthcare is to see if patients revisit EDs within short periods of time following their initial visits. For example, revisits for the same medical condition could indicate that physicians made mistakes or cut corners. To understand whether physicians who spend less practice more efficiently, I add the costs associated with ED *re*-visits to the costs associated with initial ED visits to develop total costs for “full episodes of care.” This section presents results for full episodes of care for minor injuries up to 28 days after initial ED visits, where revisits may or may not have resulted in hospitalizations.

In previous sections I estimated the results using a panel of physicians, but for data privacy reasons, I estimate the results in this section at a more aggregated level. The revisit variables come from the HCUP databases and researchers are prohibited from linking the visit-level data to identifying information about physicians. Therefore, the analysis in this section proceeds at the hospital-year-quarter-weekday-hour level. The benefit of aggregation is that the criteria for identification are less stringent and researchers in the economics of education literature have used similar methods before [26].

To make the results in this section comparable to the results in the previous section, I first residualize the outcome variables at the visit-level using the same patient characteristics that appear in Table 8. Then I aggregate the residuals to the hospital-year-quarter-weekday-hour. I also weight the regressions by the number of ED visits in each cell. Thus the estimation equation becomes,

$$\begin{array}{ccc} \hat{Y_{ht}} & = & \alpha +\psi P_{ht}+\gamma T_{t}+\delta_{h}+\epsilon_{ht} \end{array}$$ (5)

where $\hat{Y_{ht}}$ includes either the adjusted average costs for the initial visit, the adjusted 14-day or 28-day revisit rates for the same injury, or the adjusted average costs for the initial visit plus the costs for revisits for the same injury that occur within 14 or 28 days, depending on the specification. *P*_*ht*_ are the average characteristics of the physicians on staff in hospital *h* in year-quarter-weekday-hour *t*. *δ*_*h*_ is a hospital fixed effect and *T*_*t*_ includes year×quarter, weekday, and shift fixed effects.

Eq (5) asks, if the share of ED physicians with less than two years of experience increases from 0 to 1, does the emergency department revisit rate increase? Similarly, if an “episode of care” includes an initial visit plus any revisit within 14 or 28 days for the same injury, do the total costs for episodes of care increase when the share of physicians with less than two years of experience increases? If the answers are yes, then physicians who spend more are not necessarily providing better quality care.

Results for Eq (5) appear in Table 9. The first column recreates the first column from Table 8, but at the aggregated level presented in Eq (5). As the share of physicians with less than two years of experience increases from 0 to 1, average spending increases by 10.6% ($71.62/$674) and the result is statistically significant at 5% level. Columns 2 and 4 show that as the share of physicians with less than two years of experience increases, revisit rates do not change in a statistically significant way, which means that higher spending does not reduce ED revisit rates. Consistent with these findings, costs for full episodes of care (initial visit + revisits) in columns 3 and 5 are 10% higher when the share of physicians with less than two years of experience increases from 0 to 1. The results suggest that physicians who spend the most on full episodes of care are the least efficient at treating minor injuries. Nevertheless, the lower bounds of the 95% confidence intervals on the experience estimates are $12.25 and $10.62 for 14- and 28-day episodes of care, respectively, so I cannot reject relatively small differences across physicians with less versus more experience. Moreover, the previous section made clear that these spending differences are not a result of innate differences in practice style across physicians with more or less experience, but rather reflect the fact that less experienced physicians have not yet sorted into work environments that match their practice styles.

**Table 9:** Physician Experience and Total Costs for Full Episodes of Care.

	Initial Visit	14-Day Episode of Care		28-Day Episode of Care	
	(1)	(2)	(3)	(4)	(5)
	Adjusted Total Costs	Adjusted Revisit Rate	Adjusted Total Costs	Adjusted Revisit Rate	Adjusted Total Costs
<2 Years of Experience	$71.62*	0.0002	$70.97*	-0.0002	$69.48*
	(29.38)	(0.001)	(29.96)	(0.001)	(30.03)
2–4 Years of Experience	$56.88	0.00002	$59.34*	-0.00002	$59.77*
	(29.60)	(0.0009)	(30.03)	(0.0009)	(30.25)
4–6 Years of Experience	$39.89*	0.0007	$43.07*	0.0004	$42.17*
	(18.49)	(0.0007)	(18.89)	(0.0007)	(18.98)
Dependent Variable Mean	$674	0.035	$712	0.043	$721
Hospital FEs	Y	Y	Y	Y	Y
Year×Quarter FEs	Y	Y	Y	Y	Y
Shift, Weekday FEs	Y	Y	Y	Y	Y
Patient Characteristics	Y	Y	Y	Y	Y
Physician Characteristics	Y	Y	Y	Y	Y
N	886,271	886,271	886,271	886,271	886,271
*R*^2^	0.11	0.02	0.21	0.02	0.21

Notes: The dependent variables include the adjusted total costs for initial ED visits, the adjusted probabilities of revisits for the same diagnosis within 14 or 28 days, and the adjusted total costs for initial visits plus any costs associated with 14-day or 28-day revisits. Each column presents estimates from a separate regression. The sample includes ED visits for all patients with minor injuries in Florida from 2005 to 2011. The unit of analysis is the hospital-year-quarter-weekday-hour. All regressions control for hospital fixed effects, year×quarter fixed effects, weekday fixed effects, shift fixed effects, and average physician characteristics. Outcome variables are adjusted using patient age, gender, race, ethnicity, and health insurance. Standard errors are clustered at the hospital-level and are reported in parentheses.

*p < 0.05

**p < 0.01

***p < 0.001

## Conclusion

This paper asks whether physicians in the same emergency departments treat similar patients differently. I focus on patients with minor injuries who should not receive different medical care and I find considerable variation in physician practice styles within EDs. I also find that physicians with less than six years of experience spend more per ED visit with no differences in health outcomes. The association between physician experience and practice styles, however, is driven by differential sorting between high- and low-cost physicians across EDs over time. High-cost physicians are more likely to exit EDs over time, but if they remain working in EDs over time, then they are more likely to work in higher-cost EDs.

This research has raised additional questions about physician mobility and human capital that are out of the scope of this paper. One question is why high-cost physicians exit EDs over time, and where do they go? Another question relates to the process by which physicians sort. Many hospitals contract with physician groups to staff their EDs. Sorting between ED physicians and hospitals may be the result of new contracts between hospitals and physician groups. Alternatively, physician groups may assign physicians to different EDs over time based on physician preferences. Answers to these questions may shed light on why some hospitals remain persistently higher-cost than others.
